# Carpal boss syndrome: os styloideum fused to the trapezoid

**DOI:** 10.1590/0100-3984.2015.0160

**Published:** 2017

**Authors:** Gabriel Clève Nicolodi, Cesar Rodrigo Trippia, Maria Fernanda F. S. Caboclo, Raphael Rodrigues de Lima, Wagner Peitl Miller

**Affiliations:** 1 Hospital São Vicente - Funef, Curitiba, PR, Brazil.

**Figure 1:**
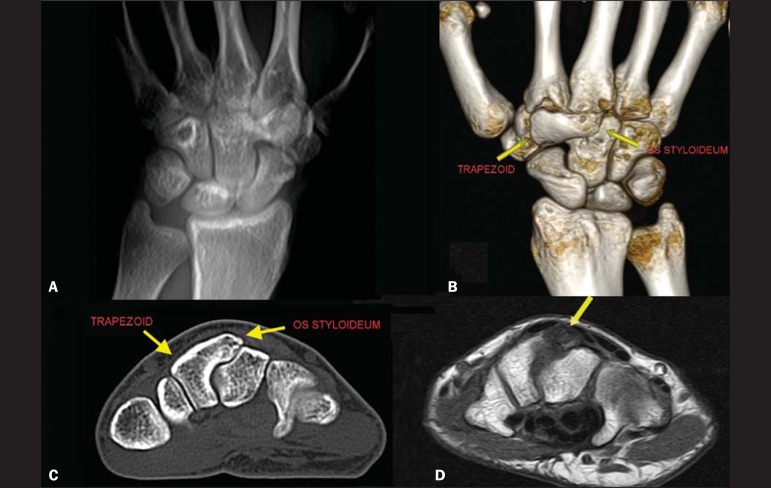
**A:** Digital X-ray showing a lack of definition of the joint space between the trapezoid and the capitate. **B,C:** Three-dimensional computed tomography reconstruction and axial computed tomography slice showing an os styloideum fused to the trapezoid and in neoarticulation with the capitate. **D:** Magnetic resonance imaging in a T1-weighted sequence, showing os styloideum with bone edema adjacent to the neoarticulation (apophysitis).

Dear Editor,

A 29-year-old White female presented with chronic pain on dorsiflexion of the right hand and a hard prominence, which was painful on palpation, at the base of the second and third metacarpal muscles. An X-ray of the hand (Figure 1A) revealed a bony prominence in the region identified as palpable in the physical examination, as well as showing that there was lack of definition of the joint space between the trapezoid and the capitate. In multiplanar and three-dimensional computed tomography reconstructions, which provided greater detail (Figures 1B and 1C), an os styloideum was seen to be fused to the trapezoid bone and in neoarticulation with the base of the third metacarpal. Magnetic resonance imaging showed a hypointense signal on a T1-weighted image (Figure 1D) and increased intensity in a T2-weighted short-tau inversion-recovery sequence, with bone edema adjacent to the neoarticulation, which is indicative of apophysitis.

Os styloideum is an anatomical variation characterized by an accessory ossicle on the dorsum of the wrist, between the trapezoid and capitate, at the base of the second and third metacarpal bones^(1)^. When it produces symptoms, mainly local pain, it is known as a carpal boss^(2,3)^. The true incidence of carpal boss syndrome is unknown; it is probably underestimated and often confused, clinically, with other causes of tumor in the dorsum of the carpus^(4)^.

Although a carpal boss can be classified as acquired (osteophytic), congenital (secondary to os styloideum), or of mixed etiology, the clinical presentations appear to be similar across the groups^(3)^. Os styloideum is also known as the ninth carpal bone^(5)^. The main difficulty in recognizing a carpal boss lies in the nonspecificity of the symptoms, which are often attributed to dorsal cysts, given that the two conditions are quite similar in terms of their location^(4)^.

The case reported here represents the rarest form of congenital carpal boss, in which the os styloideum is fused to the trapezoid, which occurs in only 0.5% of cases. More commonly (in 94.0% of cases), it is fused to the base of the second and third metacarpal, merged with the capitate (in 3.5%) or (in 2.0%) isolated^(2,6)^. The clinical presentation of carpal boss is highly variable^(2)^: the condition can be asymptomatic or can produce spontaneous pain, precipitated by excessive use of the joint or by palmar flexion of the wrist.

Knowledge of the disease and imaging studies are fundamental for the diagnosis of carpal boss and for distinguishing it from its main differential diagnoses, which include synovial cysts, fractures, osteoarthrosis, exostoses, bone neoplasms, and soft-tissue neoplasms^(7)^. Tomography studies allow the relationship between the accessory ossicle and the adjacent bones to be analyzed, and magnetic resonance imaging is important for the evaluation of the integrity of bones, entheses, and ligaments^(5)^. The proximity of the carpal boss to the short and long radial extensor tendons of the carpus can cause insertional tenosynovitis, aggravating the symptoms, especially in athletes who perform repetitive movements, specifically those involving forced flexion of the wrist ^(5,8,9)^.

The treatment for carpal boss is usually conservative, typically involving the use of anti-inflammatory drugs and, in some cases, immobilization of the wrist^(6,7)^. However, surgical excision can be required in cases that are refractory to the standard treatment^(6,7)^.
